# Collarless Polished Tapered Stems of Identical Shape Provide Differing Outcomes for Stainless Steel and Cobalt Chrome: A Biomechanical Study

**DOI:** 10.3390/jfb14050262

**Published:** 2023-05-09

**Authors:** Ayumi Kaneuji, Mingliang Chen, Eiji Takahashi, Noriyuki Takano, Makoto Fukui, Daisuke Soma, Yoshiyuki Tachi, Yugo Orita, Toru Ichiseki, Norio Kawahara

**Affiliations:** 1 Department of Orthopaedic Surgery, Kanazawa Medical University, Kahoku-gun 920-0293, Japan; 2 Department of Orthopaedics, Affiliated Renhe Hospital of China Three Gorges University, Yichang 443000, China; 3 Department of Mechanical Engineering, Kanazawa Institution of Technology, Nonoichi 921-8501, Japan

**Keywords:** bone cement, cobalt–chrome alloy, Exeter stem, periprosthetic femoral fractures, polished tapered stem

## Abstract

Cemented polished tapered femoral stems (PTS) made of cobalt–chrome alloy (CoCr) are a known risk factor for periprosthetic fracture (PPF). The mechanical differences between CoCr-PTS and stainless-steel (SUS) PTS were investigated. CoCr stems having the same shape and surface roughness as the SUS Exeter^®^ stem were manufactured and dynamic loading tests were performed on three each. Stem subsidence and the compressive force at the bone–cement interface were recorded. Tantalum balls were injected into the cement, and their movement was tracked to indicate cement movement. Stem motions in the cement were greater for the CoCr stems than for the SUS stems. In addition, although we found a significant positive correlation between stem subsidence and compressive force in all stems, CoCr stems generated a compressive force over three times higher than SUS stems at the bone–cement interface with the same stem subsidence (*p* < 0.01). The final stem subsidence amount and final force were greater in the CoCr group (*p* < 0.01), and the ratio of tantalum ball vertical distance to stem subsidence was significantly smaller for CoCr than for SUS (*p* < 0.01). CoCr stems appear to move more easily in cement than SUS stems, which might contribute to the increased occurrence of PPF with the use of CoCr-PTS.

## 1. Introduction

For cementless total hip arthroplasty (THA), the femoral stem is typically made of a titanium alloy due to its ease of processing, good biocompatibility, and minimal stress shielding. In contrast, cemented stems have been fabricated using various metal materials, such as titanium alloy, stainless steel (SUS), cobalt–chromium alloy (CoCr), among others. In the cemented stems, collarless polished tapered stems (PTS) having a surface roughness of less than 0.1 μm provide long-term results superior to stems with a surface roughness of 2.5 μm to 12 μm [1,2,3,4]. However, several studies have shown a greater frequency of postoperative periprosthetic fractures (PPF) with PTS than with composite beam-type stems [5,6,7,8]. Importantly, national joint registries have reported a higher PPF revision rate for PTS made of cobalt–chromium alloy (CoCr) than for PTS of stainless steel (SUS) [9,10].

Differences in stem design and surface roughness are thought to affect the frequency of PPFs, but insufficient data exist to confirm uniform in-cement stem behavior across stems fabricated from differing materials.

In a biomechanical study on differences between CoCr and SUS using cone rods, the researcher prepared cylindrical polished tapered rods of the same shape and having the same coefficient of friction. When the rods were fixed into cement and placed under load, the CoCr rods slipped significantly farther than the SUS rods, and the strain gauge on the cement surface around the CoCr rods demonstrated more cement creep than around the SUS rods [11]. That experiment pointed to differences in rod subsidence and cement stress between polished SUS and CoCr rods, but provided no information about differences in biomechanical behavior among stem materials in cemented stems. This experiment suggests that there may be differences in subsidence and stress on the cement between polished SUS and CoCr stems. If the CoCr-PTS exhibits greater subsidence than the SUS-PTS, this could explain the difference in PPF fracture frequency.

The Exeter^®^ stem (Stryker, Allendale, NJ, USA) is made of SUS and is the most widely used PTS in the world, providing excellent clinical results [12]. Therefore, a materials comparison between a CoCr product and the Exeter stem could be highly meaningful.

For this study, original Exeter stems made of SUS were compared to copied Exeter stems made of CoCr with the exact same shape and surface roughness, which exhibited different mechanical behaviors.

## 2. Materials and Methods

### 2.1. Implant Preparation

**Figure 1:**
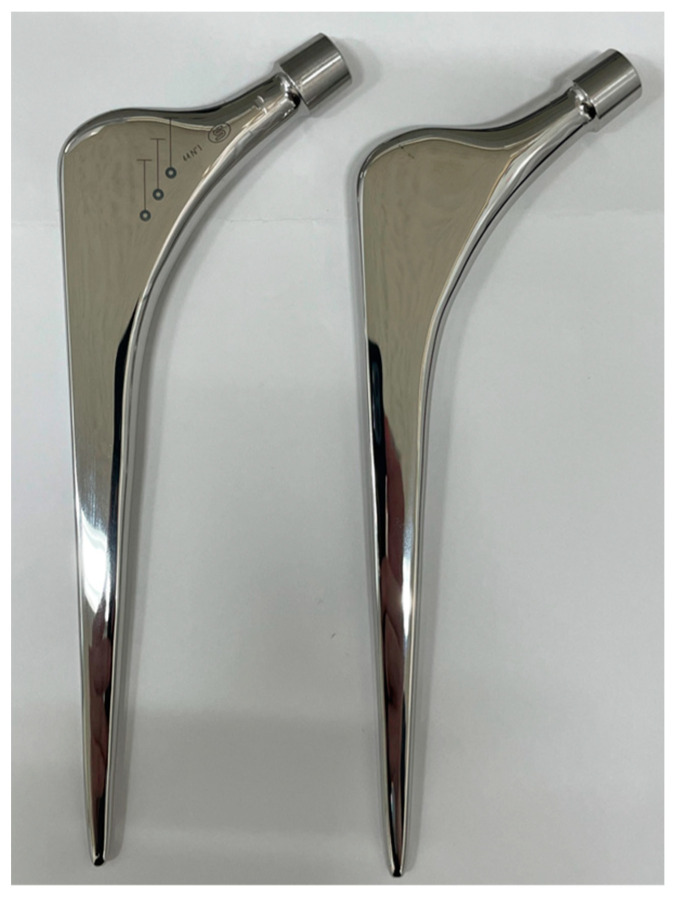
Exeter stem (SUS) and Exeter-shaped CoCr stem. On the left is an original Exeter stem. On the right is our Exeter-shaped stem of CoCr. The Exeter-shaped CoCr stem was made using the exact same design and surface roughness as the original stem.

**Figure 2:**
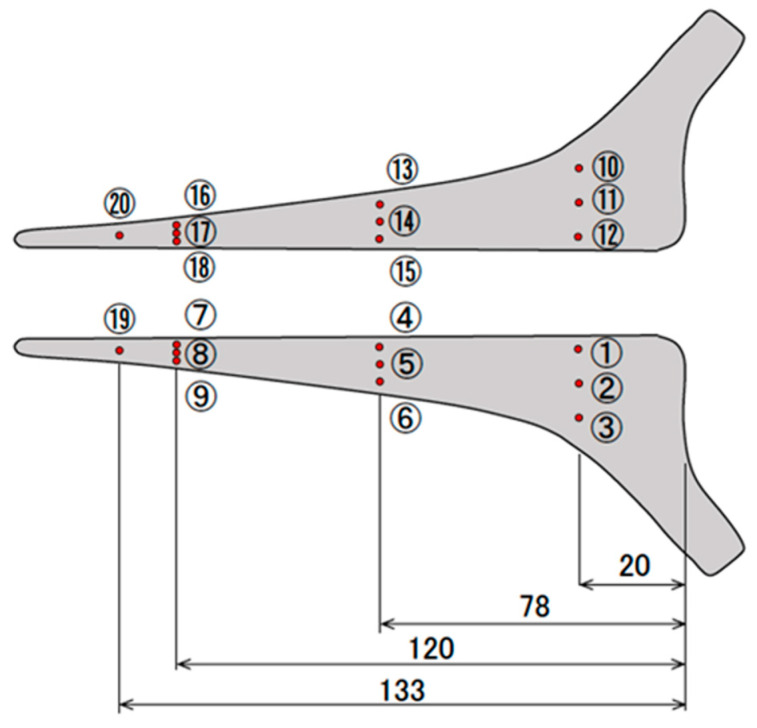
Measurement of stem surface roughness. Prior to the loading experiment, the surface roughness of the stem was analyzed. Using a laser microscope (VK-8700, Keyence, Osaka, Japan), surface roughness was assessed at 18 sites on the front and back surfaces of the stem: three positions at 20 mm (proximal), 78 mm (middle), and 120 mm (distal) from the top of the stem.

The original Exeter^®^ stem was made of SUS, but no CoCr counterpart was available. At our request, Stryker company approved this study and provided original computer-aided design (CAD) data on the Exeter stem, size 1 with 44 mm offset, for experimental purposes limited to this study. A CoCr stem identical in shape to the Exeter stem was then manufactured (Nagumo Manufacturing, Jyoetsu, Niigata, Japan) using the CAD data provided by Stryker. The manufacturing error was ±1 μ [13]. The surface of each CoCr stem was ground to a polished surface roughness of less than 0.1 µm (Yoshida Seiko, Kanazawa, Ishikawa, Japan). For this study, three Exeter (SUS) stems were prepared, and three CoCr stems with the same shape and surface finish as the Exeter stems were also prepared (Figure 1). Prior to the loading experiment, the surface roughness of the stems (one original Exeter stem and all three CoCr-Exeter-shaped stems) was measured (Figure 2). 

### 2.2. An Experimental Device

**Figure 3:**
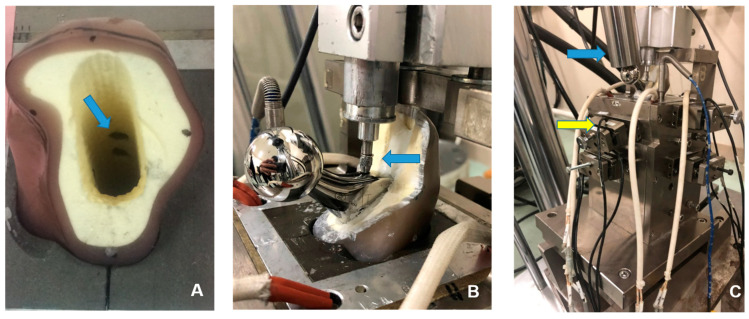
The experimental device. A unit consisting of the stem, cement, and composite femur is placed in the tester to simulate the internal human body conditions as closely as possible. (**A**) A rod (blue arrow) passed through the composite femur from the outside of the fixator. A load cell sensor to sense the pressure was placed at the bone–cement interface through this hole. (**B**). After a stem was fixed with cement into the composite femur, the digital gauge (blue arrow) was in contact with the shoulder of the stem. (**C**). The loading element was inclined at 15° with the front portion raised, so that the load was applied 15° medially to the femoral head at the top of the stem (blue arrow). The yellow arrow indicates the load cell on the proximal medial side.

The equipment for load testing and the data measurement methods used in this study were consistent with those previously reported [14,15,16]. Composite femurs were used instead of cadaveric femurs to avoid variations based on individual differences in bone strength and canal shape. The composite femurs (#3403^®^, Pacific Research Laboratories, Vashon, WA, USA) had mechanical properties comparable to the human femur. We cut the composite femur neck obliquely at 20 mm distal to the top of the greater trochanter, the same procedure as for THA, and we cut the distal portion of the femur 230 mm from the top of the greater trochanter. To create the composite femoral canal, we used an Exeter stem broach that was attached to a test fixator of S45C structural carbon steel and epoxy resin (Devcon B, ITW Industry, Osaka, Japan). Both the fixator and the composite femur were penetrated by the rod that protected the measuring equipment. This allowed us to conduct measurements at a constant site. The rod passing through the inner tube was secured on the face of the medullary canal of the composite femur (Figure 3A). To mimic the wet conditions of the in vivo femoral environment, the composite femurs were soaked in blended vegetable oil for 24 h before attaching them to the test equipment. An 80 g sample of bone cement (Simplex p, Stryker, NJ, USA) was mixed in a vacuum cement mixing system (ACM vacuum mixing ball, Stryker, Tokyo, Japan), and a cement injection gun was used to insert the mixture into the femoral canal. Based on reports that the creep and mechanical behavior of bone cement can differ significantly between room temperature and body temperature [17], the experiment was performed to mimic in vivo environmental conditions by maintaining the test equipment at 37 °C with a temperature sensor (T-35^®^, Takigen MFG Co., Ltd., Tokyo, Japan) and a heater (G6A92^®^ [240 V, 250 W], Takigen MFG Co., Ltd., Tokyo, Japan). 

### 2.3. Stem and Tantalum Ball Insertion

After the cement was delivered, each stem was inserted into the composite femur. Before the cement hardened, we used an indwelling needle to inject tantalum balls 0.6 mm in diameter into the cement around the stem. Before and after the loading test, micro-computed tomography (CT) was taken from the area of the neck cut to 30 mm below the lesser trochanter, to measure the three-dimensional movement of each tantalum ball. The movement of the tantalum ball was assumed to represent the movement of the cement around the stem. Measurements were performed according to the method described by Takahashi et al. [15]. The movement of the balls was measured both horizontally and vertically, independent of the stem, and the final amount of subsidence for each stem and the ratios of ball subsidence/stem subsidence were calculated.

### 2.4. Loading Method and Loading Period

Cyclical loading (1 Hz, 30 to 3000 N) was applied 500,000 times to a metal femoral head (cobalt–chromium alloy, 26 mm in diameter) attached to the stem neck. A fatigue testing system (EHF-UM 300KN-70L; Shimadzu Corporation, Kyoto, Japan) was used at an angle of 15° medially to the coronal plane of the model to mimic normal in vivo loading in a 70 kg adult [18]. In this experiment, an eight-hour non-loading period was incorporated between the 16 h loading periods to simulate the actual sleep period in a clinical setting and allow for stress relaxation of the cement.

### 2.5. Measurement of Stem Subsidence

A digital displacement gauge (DTH-A-5, 5 mm; Kyowa Electronic Instruments Co., Ltd., Tokyo, Japan, Figure 3B) was placed on the proximal lateral portion of the stem to measure stem subsidence over time. Data were transmitted automatically to a computer through data collection and analysis software (Sensor Interface PCD-300A, Kyowa Electronic Instruments Co., Tokyo, Japan). Due to the vast amount of collected data, a single file containing 8 min of measurement data every hour was used, and 216 files (24 h a day for 9 days) were generated for each loading test. The 16 h loading period for each day was divided into early, middle, and late phases, and the first two files from each phase (a total of 20,000 values) were collected for use. Subsidence was defined as the mean of the maximum values for sine waves from the 20,000 values during each phase of the loading period. Additionally, the mean values of the 20,000 data sets were collected for the duration of non-loading. On the final day of the experiment, the final compressive force and stress relaxation values were collected as the mean and standard deviation (SD) values. Graphs were made for continuous data on stem subsidence for all models throughout the experiment. 

### 2.6. Measurement of Compressive Force at the Bone–Cement Interface

After the cement had hardened, a rod in the tube from outside the fixator was connected to a load cell (TR20I 500N/fs^®^, TR20I 200N/fs^®^, Kyowa Electronic Instruments Co., Ltd., Tokyo, Japan, Figure 3C) and the pressure transducer. The rod was placed at eight sites on the medial, lateral, anterior, and posterior sides of the proximal and distal femur at the interface with the bone cement in the femoral canal. We measured compressive forces at the cement–bone interface over time after calibration and input those measured values automatically into a computer. The loading and non-loading periods each day were classified into 3 periods (early, middle, and late). The compressive force in each period was defined as the mean of the collected 960 maximum values of sine waves in the 2 consecutive files after the start of that period (60 values/min × 8 min × 2 times). In total, 27 averaged values (3 × 9 days) were used to analyze stem subsidence and compressive force, respectively. Continuous data of compressive force were graphed throughout the experiment.

### 2.7. Measurement of Cement Thickness

**Figure 4:**
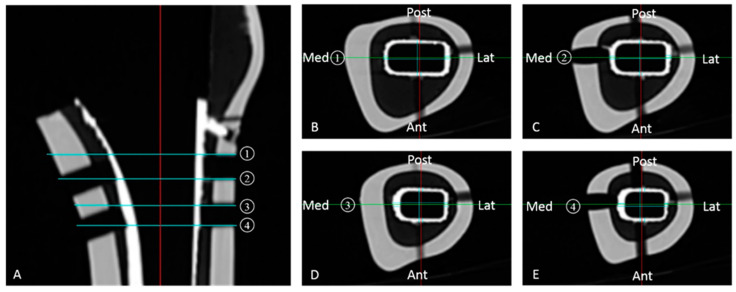
CT images, taken after stem removal. CT images were taken at 1 mm slice spacing in four horizontal sections aligned with the insertion holes of the proximal lateral femoral rods to measure the cement thickness. (**A**) Coronal slice of femoral canal center. 1: Upper edge of proximal lateral hole for rod. 2: Lower edge of proximal lateral hole for rod. 3: Upper edge of distal lateral hole for rod. 4: Lower edge of distal lateral hole for rod. (**B**) Axial slice at the level of 1; (**C**) Axial slice at the level of 2; (**D**) Axial slice at the level of 3; (**E**) Axial slice at the level of 4. The thickness of the cement layer was measured at the midpoint of each area.

After the loading tests were completed, all stems were removed from the cement. CT images were then taken with a slice spacing of 1 mm to measure the thickness of the cement mantle. The average thickness of the cement was calculated using four scans of horizontal sections aligned with the insertion holes of the proximal rods. The thickness of the cement layer was measured in the middle of each of the anterior, posterior, medial, and lateral areas (Figure 4).

### 2.8. Statistical Analysis

We compared the amount of stem subsidence, compressive force at the bone–cement interface, tantalum ball behavior, and surrounding bone cement thickness between the two groups using the Mann–Whitney U test. The relationship between stem subsidence and compressive force at the bone–cement interface was analyzed using Pearson’s correlation coefficient. Statistical significance was indicated for a probability (alpha) of 0.01. All statistical analyses were performed using the Social Science Package version 18.0 (SPSS Inc., Chicago, IL, USA).

## 3. Results

### 3.1. Surface Roughness of the Stem before the Experiment

Stem surface roughness was measured before the load test was initiated. The surface roughness of the Exeter stem and the CoCr stems was Ra = 49.61 (SD 13.59) nm and Ra = 52.56 (SD 9.32) nm, respectively. The surface roughness for all areas of all polished stems was less than 100 nm (=0.1 μm). There were no obvious differences in surface roughness across all areas and all stems (Table 1).

### 3.2. The Amount of Stem Subsidence (Figure 5)

Subsidence was greatest on the first day, followed subsequently by gradual subsidence in both stem models. Mean final subsidence was significantly greater in the CoCr group, at 0.478 (SD 0.063) mm vs. 0.257 (SD 0.094) mm in the SUS group (*p* < 0.001). During the loading period, first-day subsidence was 0.123 (SD 0.046) in the SUS group and 0.138 (SD 0.056) in the CoCr group (*p* = 0.355), and first-day rise was 0.114 (SD 0.035) and 0.131 (SD 0.050), respectively (*p* = 0.323), with no significant difference between the two groups in either subsidence or rise during the first day. Excluding the first day, stem subsidence was significantly greater in the CoCr group, at 0.1028 (SD 0.0207) mm vs. 0.0437 (SD 0.0203) mm in the SUS group (*p* < 0.001). Stem rise throughout the non-loading period was greater for CoCr, at 0.0674 (SD 0.0153) mm than 0.0295 (SD 0.0148) mm for SUS (*p* < 0.001).

**Figure 5:**
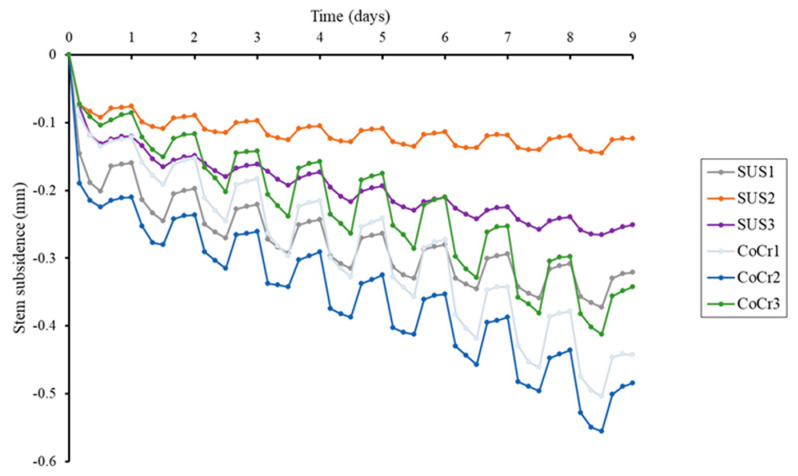
Diagram showing the subsidence of stems. All stems subsided rapidly on the first day and then gradually continued to subside. All stems subsided during loading and rose during non-loading. Except for the first day, daily stem subsidence and stem rise was significantly greater for the CoCr stems than for the SUS stems. Final subsidence of the three CoCr stems was significantly greater than that of the three SUS stems.

### 3.3. Compressive Force at the Bone–Cement Interface

**Figure 6:**
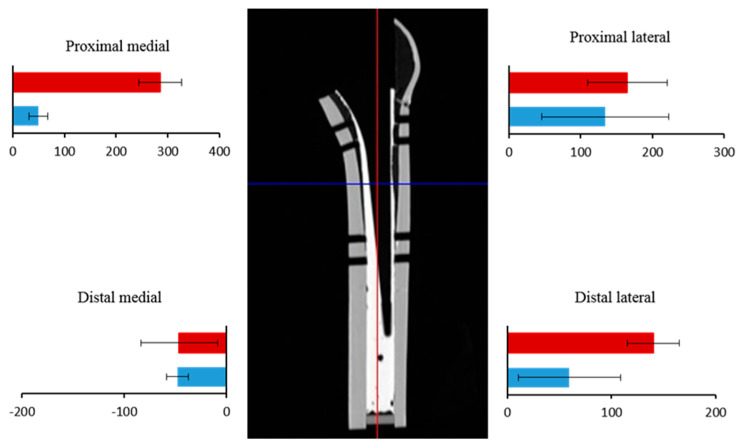
Final forces at the cement–bone interface. Final compressive forces are shown for the SUS (blue) and CoCr (red) stems. The three major regions of compressive force were the proximal medial and lateral regions and the distal lateral region. The bars show mean force in each group, and the lines show standard deviation. The forces differed significantly between the two groups in the proximal medial and distal lateral regions. (Proximal medial *p* value < 0.001; distal medial *p* value = 0.258; proximal lateral *p* value = 0.258; distal lateral *p* value = 0.004).

The final maximum compressive force at the bone–cement interface for both stems was measured for four stems (SUS1, SUS3, CoCr1, CoCr2) on the proximal medial side and for two on the proximal lateral side (SUS2, CoCr3) (Figure 6). The final compressive force was greater in the CoCr group than in the SUS group at the proximal medial (*p* < 0.001) and distal lateral (*p* = 0.004) region significantly (Figure 6). 

**Figure 7:**
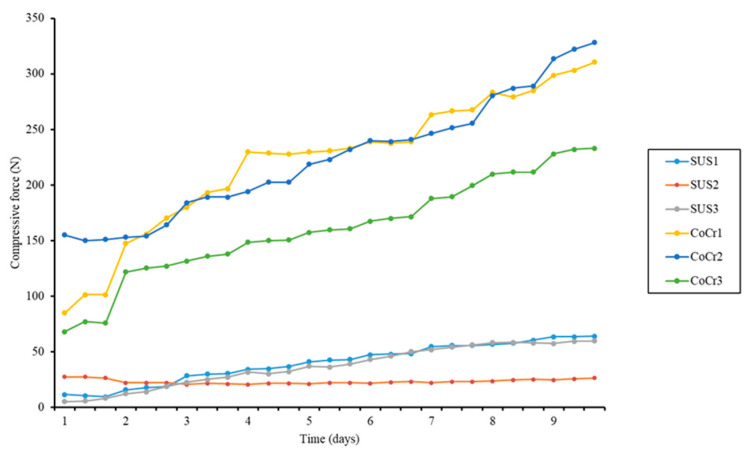
Compressive forces at cement–bone interface in proximal medial region throughout the experiment. Compressive forces from CoCr stems were much greater than from SUS stems throughout the experiment. The mean compressive force associated with CoCr was significantly greater than with SUS on the final day.

The forces in the anterior and posterior regions were small values compared to the medial and lateral regions. The compressive forces at the cement–bone interface in the proximal medial region gradually increased over the course of the experiment. The compressive forces generated by all CoCr stems were significantly greater than those generated by all SUS stems throughout the experiment, and the rate of increase in compressive forces was greater for the CoCr stems than for the SUS stems (Figure 7).

**Figure 8:**
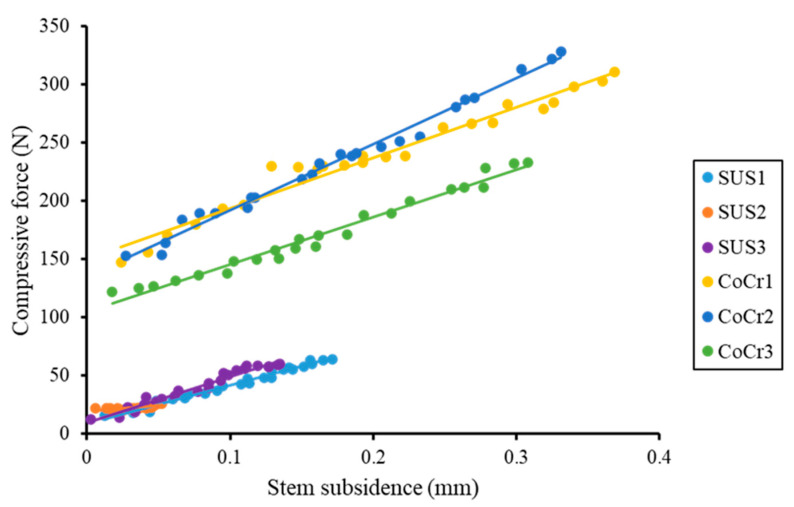
Correlation of stem subsidence and compressive force in proximal medial region. Dots represent the values of each point of stem subsidence and compressive force since the second day. An approximate straight line for each dot was calculated for each stem, and a straight line was generated.

A strongly significant positive correlation was demonstrated between subsidence values and compressive forces in all stems, indicating that the stem subsidence generated compressive force at the cement–bone interface (Figure 8). Between 0.05 mm and 0.1 mm of subsidence, the compressive force was more than four times greater for CoCr than for SUS, a significant difference (37.42 (SD 7.41) N for SUS and 166.34 (SD 23.14) N for CoCr, *p* < 0.001) and consistent with our findings for subsidence between 0.1 and 0.15 mm (53.68 (SD 5.65) N in the SUS group and 186.62 (SD 31.64) N in the CoCr group, *p* < 0.001). In other words, even with the same amount of subsidence, CoCr stems generated a larger compressive force, more than three times that for SUS stems at the bone–cement interface.

A strongly significant positive correlation was observed between stem subsidence and compressive force at the cement–bone interface in all stems. Correlation equations, *p* values, and coefficient of determination (r^2^) are shown below, from the left: 

SUS1: y = 329x + 8.7903, *p* < 0.01 (r^2^ = 0.9787)

SUS2: y = 93.348x + 19.413, *p* < 0.01 (r^2^ = 0.5432)

SUS3: y = 394.85x + 9.5199, *p* < 0.01 (r^2^ = 0.9626)

CoCr1: y = 433.82x + 150.43, *p* < 0.01 (r^2^ = 0.9668)

CoCr2: y = 567.14x + 135.41, *p* < 0.01 (r^2^ = 0.9881)

CoCr3: y = 407.12x + 104.86, *p* < 0.01 (r^2^ = 0.9775).

### 3.4. Movement of Tantalum Balls

The tantalum balls were attempted to be positioned around the stem on the medial, lateral, anterior, and posterior sides, but in some cases, it was not possible to successfully place the balls on the posterior side of the stems. As a result, the tantalum balls in the posterior cement were excluded from the analysis.

The actual distance of tantalum ball movement may vary for each composite femur due to calibration differences; therefore, the distances cannot be compared simply. For this reason, the ratio of horizontal movement to vertical movement was compared for each ball used with each femur. Although there was no difference between the SUS and CoCr stems in the ratio of horizontal/vertical ball movement (*p* = 0.863), the ratio of ball vertical movement to stem subsidence was significantly smaller for CoCr than for SUS (*p* = 0.008). 

This result indicated that CoCr stems slipped more against the cement surface than did the SUS stems, even with identical ratios for cement creep (Table 2).

### 3.5. Cement Thickness

Due to the hard cancellous bone in this composite bone model, there was minimal cement integration into the bone. As a result, the cement mantles were highly detectable and measurable with accuracy on all slices of CT in all composite femurs.

Between the two groups, there was no significant difference in cement thickness around the lesser trochanter between the SUS and CoCr groups (mean thickness 1.813 mm for SUS and 1.842 mm for CoCr) (Table 3). Results also showed no difference in stem alignment between the two groups.

## 4. Discussion

Different alloy materials exhibiting different mechanical behaviors have been investigated by researchers. Takegami et al. [19] performed biomechanical tests on the CPT, VerSys Advocate, and CMK stems, and noted that the median fracture torque for the CPT stem was significantly lower than for the CMK stem. They suggested that variations in the mechanical behavior of these prosthetic materials might contribute to differences in the incidence of PPF. 

To replicate a physiologic environment as closely as possible, our experiment employed 1 Hz sine curve loading to simulate a normal human walking pace, maintained a wet environment for the composite bone, kept the model at a temperature of 37 °C, and provided an 8 h period of stress relaxation per day. To ensure a reliable comparison between CoCr and SUS materials, stems were confirmed to have identical design and surface roughness, and were placed in cement of identical thickness. Therefore, our results were considered highly reliable.

Under these reliable conditions, significant differences were observed between CoCr and SUS stems. CoCr stems exhibited greater daily subsidence and rise, as well as greater final subsidence, suggesting that the CoCr stems were more prone to slippage within the cement compared to the SUS stems.

By investigating the movement of tantalum balls within the cement, we confirmed the movement of the cement around the stem. As a result, CoCr stems were shown to be more susceptible to slippage on the cement surface compared to SUS stems.

Previous reports have suggested that, with a polished surface of Ra = 0.06 µm and bone cement of moderate viscosity, the frictional coefficient was significantly lower for CoCr than for SUS [20], consistent with findings that CoCr had lower wettability than SUS and provided relatively poor adhesion to bone cement [11,20]. This would explain the greater mobility of CoCr stems in the cement. 

Grammatopoulos et al. [21] investigated the double taper PTS fracture type and reported that 16 of 21 clinical cases were of the Vancouver B2 type, involving spiral fractures, stem subsidence, and cement rupture. Morishima et al. [22] fixed the Exeter stem with cement in composite bone and applied internal rotation while loading the prosthesis, resulting in type B2 spiral fractures in all cases. The Vancouver type is considered to be the most common clinical PPF morphology associated with PTSs, and these findings indicate that stem subsidence with rotational force is an important factor in the occurrence of PPF when using a PTS [21,22,23,24].

Stem subsidence is thought to generate compressive force at the cement–bone interface because of the strong correlation between stem subsidence and these compressive forces [14,15,16]. In this study, mean final subsidence was significantly greater for CoCr stems than for SUS (0.491 mm and 0.261 mm, respectively). Compressive forces were also significantly higher for CoCr than for SUS. Furthermore, even with equal levels of subsidence, compressive forces were found to be more than three times higher for CoCr stems than for SUS stems at the cement–bone interface. This was believed to be due to the fact that CoCr stems were more prone to slip on the cement surface compared to SUS stems, as evidenced by the results of the tantalum balls. Our findings suggest that CoCr stems exert greater pressure on the femur compared to SUS stems. This conclusion aligns with previous research indicating that significant stem subsidence may induce great hoop stress [14].

Some reports also show that, when inserted into a thicker cement layer, a PTS stem of CoCr will subside more, causing the cement surrounding the stem to be dragged in the direction of subsidence, which is toward the shear force [15,16]. The significant subsidence of the PTS in a thicker cement mantle may likewise generate high stress in the cement and predispose to PPF [15,25]. However, there was no difference in the thickness of the cement between the CoCr stem and SUS stem groups in this study. Therefore, our results, which excluded factors other than material differences, suggest that the CoCr stem, which is prone to large movements and sinking, may be more likely to cause PPF than the SUS stem.

Our study has some limitations. (1) Only a limited number of stems (three SUS and three CoCr) were used in the experiment. (2) Loading was only applied from one direction, and no real clinical rotation was added; other results might be obtained with the addition of stem rotation. (3) The composite femur used in this experiment might differ from the actual behavior of a human femur. (4) The use of different stem designs and stem cements could yield different results. (5) In contrast to cadaveric femurs, composite bone has the benefit of being highly standardized. However, composite femurs lack cancellous bone in the diaphysis area, resulting in a thick mantle (more than 3 mm) in the distal region.

Strengths of our study include the use of composite bone, which in contrast to cadaveric femurs, has the benefit of being highly standardized even though only three pairs of stems were used. Although the differences between stem brands cannot be excluded from consideration, the differences in mechanical behavior between materials are practical and quantifiable. Our findings would be expected to differ if applied to cadaver bones or to individual living organisms. However, we consider the data in this study to be as reliable as previous similar biomechanical experiments using composite bones [14,15,16].

## 5. Conclusions

Our findings clearly demonstrated that the Exeter-shaped CoCr stem showed greater subsidence in the cement and higher bone–cement interface forces than the original Exeter stem made of SUS. Our study suggested that the CoCr stem was more prone to slipping on the cement surface than SUS and generated over three times the hoop stress of SUS with the same amount of subsidence. These differences might appear to contribute to the increased risk of PPF when using CoCr-PTS.

## Figures and Tables

**Table 1:** Surface roughness in all stem areas.

		SUS Group			CoCr Group		
		No.1	No.2	No.3	No.1	No.2	No.3
Front	proximal	50.67 (22.19)	35.00 (9.64)	49.33 (22.12)	54.00 (1.00)	38.67 (0.58)	46.33 (1.15)
	middle	41.33 (3.21)	40.00 (3.61)	43.00 (3.46)	48.33 (3.06)	49.00 (5.29)	46.33 (4.04)
	distal	58.67 (10.40)	66.33 (6.03)	54.33 (10.26)	54.33 (8.50)	55.33 (14.01)	57.33 (8.14)
Back	proximal	47.00 (1.73)	32.00 (2.65)	43.67 (2.31)	60.00 (7.21)	51.33 (6.43)	42.33 (1.52)
	middle	57.33 (10.12)	37.00 (1.00)	59.00 (10.39)	56.33 (3.21)	53.00 (5.57)	42.00 (5.29)
	distal	67.00 (8.89)	45.67 (9.02)	65.67 (9.45)	64.33 (10.50)	68.33 (7.64)	58.67 (1.53)

Surface roughness was assessed on the front and back surfaces of the stem: proximal, middle, and distal from the top of the stem as shown in Figure 2. Numbers indicate mean thickness in each group. Numbers within ( ) are standard deviation. The notation unit is nm.

**Table 2:** There were no significant differences in the horizontal/vertical distance ratio of balls between the SUS and CoCr groups. However, the ratio of vertical distance/stem subsidence was significantly smaller in the CoCr group than in the SUS group.

	SUS1			SUS2			SUS3			CoCr1			CoCr2			CoCr3			*p* Value
Position of Ball	Medial	Lateral	Anterior	Medial	Lateral	Anterior	Medial	Lateral	Anterior	Medial	Lateral	Anterior	Medial	Lateral	Anterior	Medial	Lateral	Anterior	
Number of balls	1	1	3	1	1	3	1	1	3	1	2	2	1	1	2	1	1	2	
Stem subsidence (mm)	0.36527			0.14203			0.26417			0.49105			0.54482			0.39862			
Horizontal/Vertical movement of ball (A)	0.0926	0.7883	0.5541	0.0811	0.3944	0.2513	0.0244	0.6087	0.1162	0.1077	2.0332	0.1863	0.1667	0.3774	0.1130	0.0515	5.5714	0.3562	
Vertical movement of ball/ Stem subsidence (B)	0.4435	0.4184	0.2378	1.0420	0.5622	0.7456	0.1552	0.0871	0.1313	0.3971	0.0697	0.1445	0.0661	0.0973	0.0631	0.2433	0.0176	0.0609	
Mean ± SD (A)	0.3235 (0.2751)									0.9959 (1.8237)									0.863
Mean ± SD (B)	0.4248 (0.3184)									0.1288 (0.1199)									0.008

**Table 3:** Thickness of cement in each CT slice. Numbers indicate mean thickness in each group. Numbers within ( ) are standard deviation. The notation unit is nm.

	SUS Group				CoCr Group			
	Medial	Lateral	Anterior	Posterior	Medial	Lateral	Anterior	Posterior
No.1	2.23 (0.334)	1.95 (0.250)	1.73 (0.179)	1.38 (0.083)	2.28 (0.130)	2.1 (0.187)	1.63 (0.148)	1.33 (0.083)
No.2	1.98 (0.192)	2.03 (0.148)	1.5 (0.071)	1.28 (0.083)	2.38 (0.083)	2.23 (0.109)	1.53 (0.109)	1.6 (0.071)
No.3	2.38 (0.130)	2.28 (0.130)	1.55 (0.112)	1.50 (0.122)	2.10 (0.158)	2.13 (0.311)	1.53 (0.148)	1.30 (0.071)

## Data Availability

The data presented in this study are available on request from the corresponding author.
